# Why spatial is special in education, learning, and everyday activities

**DOI:** 10.1186/s41235-021-00274-5

**Published:** 2021-03-23

**Authors:** Toru Ishikawa, Nora S. Newcombe

**Affiliations:** 1 INIAD Toyo University, Tokyo, Japan; 2 Temple University, Philadelphia, USA

The structure of human intellect can be conceptualized as consisting of three broad but correlated domains: verbal ability, numerical ability, and spatial ability (Wai et al. 2009). Verbal and numerical abilities are traditionally emphasized in the classroom context, as the phrase "the three Rs" (reading, writing, and arithmetic) suggests. However, research has increasingly demonstrated that spatial ability also plays an important role in academic achievement, especially in learning STEM (science, technology, engineering, and mathematics) (National Research Council 2006; Newcombe 2010). For example, envisioning the shape or movement of an imagined object contributes to the understanding of intersections of solids in calculus, structures of molecules in chemistry, and the formation of landscapes in geology.

*Spatial thinking* is a broader topic than spatial ability, however (Hegarty 2010). We use *symbolic spatial tools,* such as graphs, maps, and diagrams, in both educational and everyday contexts. These tools significantly enhance human reasoning, for example, graphs are a powerful tool to show the relationship among a set of variables in two (or higher) dimensions. STEM disciplines use these tools frequently, and, in addition, often have specific representations that students need to master, such as block diagrams in geology. Although teachers may assume that these representations are easy to read, maps, diagrams and graphs often pose difficulty for students, especially those with low spatial ability (e.g., a graph that shows changes in an object's velocity according to time) (Kozhevnikov et al. 2007).

As well as understanding spatial representations that are provided by teachers or in textbooks, good spatial thinkers can choose or even create representations that are suitable for the task at hand. Novices tend to prefer representations that are realistic and detailed, often more realistic and detailed than necessary because they include irrelevant information (Hegarty 2010; Tversky and Morrison 2002). Being good at spatial thinking entails the ability to select and create appropriate spatial representations, based on sound knowledge of content in a specific domain.

Navigation is a special kind of spatial thinking, which requires us to understand our location (where we are) and orientation (which direction we are facing) in relation to the surroundings. Sometimes, we may construct reasonably accurate mental representations of the environment ("maps in the head" or "cognitive maps"). However, people often have difficulty with cognitive mapping (Ishikawa and Montello 2006; Weisberg and Newcombe 2016), especially in environmental space (beyond figural or vista space), when we cannot view a layout in its entirety from a single viewpoint (Ittelson 1973; Jacobs and Menzel 2014; Montello 1993). People thus need to move around and integrate separate pieces of information available at each viewpoint in a common frame of reference, which poses extra cognitive processing demands (Han and Becker 2014; Holmes et al. 2018; Meilinger et al. 2014). Spatial orientation and navigation may be problematic for some people even with maps or satellite navigation (Ishikawa 2019; Liben et al. 2002).

## Characteristics of spatial thinking

Spatial thinking has unique characteristics that offer interesting research challenges. First, spatial thinking concerns space at different scales. Thinking about the structures of molecules, envisioning the folding and unfolding of a piece of paper, making a mechanical drawing, packing a suitcase, finding your way to a destination in a new environment, and reasoning about the formative process of a geologic structure all concern thinking and reasoning about space, but they span a wide range of spatial and temporal scales. Expertise in spatial thinking in STEM domains typically focuses on a specific scale, with organic chemistry, surgery, mechanical engineering, architecture, structural geology, and planetary science spanning but not exhausting the range. Spatial skills may vary across scale. For example, Hegarty et al. (2006) showed that learning from direct navigation in the environment differed from learning from a video or a desktop virtual environment, yielding two separate factors in factor analysis, and that the former was correlated with self-report sense of direction, whereas the latter with psychometrically assessed spatial ability. Learmonth et al. (2001) showed that young children's use of landmark information to reorient depends on the size of space.

Second, spatial thinking occurs in various media, including 2D static images, 3D animations, schematic diagrams, indoor and outdoor environments, immersive virtual environments, and spatial language. Each medium has its own way of representing spatial information (Liben 1999; Tversky 2001) and knowledge acquired from different media differs in structure and flexibility in important ways (Rieser 1989; Taylor and Tversky 1992; Thorndyke and Hayes-Roth 1982). In discussing spatial thinking and learning media, one should distinguish between internal representations (knowledge in the mind) and external representations (spatial products or expressions presented to a person). External spatial representations are shown visually in a certain level of detail or resolution (Goodchild and Proctor 1997), and verbally in a specific frame of reference (Levinson 1996).

Third, spatial thinking skills vary both at a group level and at the individual level. There are cases where group differences are of concern to the instructor, for example, in consideration of male–female differences in entry and retention rates in STEM disciplines (Belser et al. 2018; Chen 2013; Sithole et al. 2017). Instructors are also concerned with individual differences in aptitudes; for example, students vary in their spatial and verbal abilities and some students are good at spatial tasks and some are good at verbal tasks. Is there a good way to adjust instructional methods to students' aptitudes? Furthermore, given the existence of group and individual differences in spatial thinking, another question of concern is how instruction can have an impact, for example, whether male–female differences in spatial thinking, when they occur, can be eliminated, or how best people with difficulty in spatial thinking can improve, by training.

## Papers in this special issue

The papers in this special issue center around three major topics: (a) spatial thinking and the skill of mental rotation; (b) spatial thinking in the classroom context or in STEM curricula; and (c) spatial thinking in wayfinding or large-scale spatial cognition. Here is a link to the papers (https://cognitiveresearchjournal.springeropen.com/spatial-collection) (Table 1).

**Table 1:** Papers in this special issue

*1. Spatial Thinking and the Skill of Mental Rotation*
1.1. Spatial thinking in infancy: Origins and development of mental rotation between 3 and 10 months of age Scott P. Johnson and David S. Moore
1.2. Strengthening spatial reasoning: Elucidating the attentional and neural mechanisms associated with mental rotation skill development Katherine C. Moen, Melissa R. Beck, Stephanie M. Saltzmann, Tovah M. Cowan, Lauryn M. Burleigh, Leslie G. Butler, Jagannathan Ramanujam, Alex S. Cohen, and Steven G. Greening
1.3. Spatial anxiety mediates the sex difference in adult mental rotation test performance Daniela Alvarez-Vargas, Carla Abad, and Shannon M. Pruden
1.4. Telling right from right: The influence of handedness in the mental rotation of hands You Cheng, Mary Hegarty, and Elizabeth R. Chrastil
1.5. Spatial ability contributes to memory for delayed intentions Veit Kubik, Fabio Del Missier, and Timo Mäntylä
*2. Spatial Thinking in the Classroom Context or in STEM Curricula*
2.1. Situating space: Using a discipline-focused lens to examine spatial thinking skills Kinnari Atit, David H. Uttal, and Mike Stieff
2.2. Gesture during math instruction specifically benefits learners with high visuospatial working memory capacity Mary Aldugom, Kimberly Fenn, and Susan Wagner Cook
2.3. When it all falls down: The relationship between intuitive physics and spatial cognition Alex Mitko and Jason Fischer
2.4. Focus on the notice: Evidence of spatial skills’ effect on middle school learning from a computer simulation Colleen M. Epler-Ruths, Scott McDonald, Amy Pallant, and Hee-Sun Lee
2.5. Unpacking the black box of translation: A framework for infusing spatial thinking into curricula Kristin M. Gagnier and Kelly R. Fisher
2.6. Elementary teachers’ attitudes and beliefs about spatial thinking and mathematics Heather Burte, Aaron L. Gardony, Allyson Hutton, and Holly A. Taylor
*3. Spatial Thinking in Wayfinding or Large-Scale Spatial Cognition*
3.1. Reference frames in spatial communication for navigation and sports: An empirical study in ultimate frisbee players Steven M. Weisberg and Anjan Chatterjee
3.2. Exploring the effects of geographic scale on spatial learning Jiayan Zhao, Mark Simpson, Jan Oliver Wallgrün, Pejman Sajjadi, and Alexander Klippel
3.3. Where are we going and where have we been? Examining the effects of maps on spatial learning in an indoor guided navigation task Mallory C. Stites, Laura E. Matzen, and Zoe N. Gastelum
3.4. Improving cognitive mapping by training for people with a poor sense of direction Toru Ishikawa and Yiren Zhou
3.5. Uncertainty promotes information-seeking actions, but what information? Ashlynn M. Keller, Holly A. Taylor, and Tad T. Brunyé
3.6. Spatial activity participation in childhood and adolescence: Consistency and relations to spatial thinking in adolescence Emily Grossnickle Peterson, Adam B. Weinberger, David H. Uttal, Bob Kolvoord, and Adam E. Green
3.7. Childhood wayfinding experience explains sex and individual differences in adult wayfinding strategy and anxiety Vanessa Vieites, Shannon M. Pruden, and Bethany C. Reeb-Sutherland

### Mental rotation

Mental rotation is one of the major spatial abilities assessed by psychometric spatial tests, and has been much studied. Importantly, it has been shown to correlate with success in a variety of other spatial thinking tasks. Intriguingly, it also shows large male–female differences in adults, although sex differences in other spatial skills tend to be smaller or even non-existent. Whether there are sex differences in mental rotation in children is a more controversial topic; sex differences may emerge over the course of development (Lauer et al. 2019; Newcombe 2020), but for an alternative, see Johnson & Moore’s paper in this special issue. There are also papers in the special issue investigating the malleability of mental rotation with practice (Moen et al.), and its relations with spatial anxiety (Alvarez-Vargas, Abad, & Pruden) and everyday experience (Cheng, Hegarty, & Chrastil). In an unexpected twist, it turns out that mental rotation may even be involved with tracking tasks and executing intended actions at specified times (Kubik, Del Messier, & Mantyla).

### Spatial thinking in STEM

Spatial thinking, as discussed above, includes advanced disciplinary thinking of a spatial nature, based on expert knowledge and reasoning in each domain. Examples of such academic disciplines include structural geology, surgery, chemistry (Atit, Uttal, & Stieff), and mathematics (Aldugom, Fenn, & Cook). Despite the contribution of spatial thinking to a physical prediction task, however, spatial skills did not account for all of the individual differences observed in intuitive physics (Mitko & Fischer). Variation in spatial learning is already evident in early adolescence, as shown in a study of learning about plate tectonics using a computer visualization (Epler-Ruths, McDonald, Pallant, & Lee). The development of effective spatial instruction should consider how to bring scientific research into the educational practice of spatial thinking (Gagnier & Fisher) and how to support elementary school teachers who are liable to spatial anxiety (Burte, Gardony, Hutton, & Taylor).

### Spatial thinking and navigation

Space at environmental scale, or navigational spatial thinking, is vital in everyday life for wayfinding in the environment. Issues of concern to researchers include spatial reasoning in different spatial frames of reference (Weisberg & Chatterjee), learning performance at different spatial scales (Zhao et al.), relationship with sense of direction (Zhao et al.; Stites, Matzen, & Gastelum), the possibility of improving cognitive mapping skills (Ishikawa & Zhou), and navigation in complex environments or emergent situations (Stites, Matzen, & Gastelum). Uncertainty in a novel environment prompts people to seek information, and a review of the literature suggests the importance of examining task behavior, not just the state of knowledge at the end of a navigation experience (Keller, Taylor, & Brunye). In the context of a discussion of the possibility of instructing spatial thinking, participation in spatial activities during childhood or adolescence and its relationship with spatial thinking has attracted the attention of researchers and practitioners (Peterson et al.). Sex differences in navigation may arise from girls and boys having different childhood wayfinding experiences (Vieites, Pruden, & Reeb-Sutherland).

## Questions for further thinking about spatial thinking

Looking over the articles in the special issue as well as other recent studies suggests questions for further research into spatial thinking.

### Spatial ability and spatial thinking

How does mental rotation relate to spatial thinking in various academic disciplines? The existing literature points to the malleability of the skill of mental rotation: given that mental rotation is an important component of spatial thinking, how can training in mental rotation improve (or transfer to) spatial thinking? Does the effect differ in different disciplines or for different types of spatial thinking in a specific discipline? What about examining other spatial abilities, such as perspective taking, spatial orientation, or flexibility of closure, in regard to their relations with spatial thinking of various kinds? Arguably, we have focused too much on mental rotation, and ignored other kinds of crucial mental operations.

### Spatial thinking as a domain-specific learning skill

Researchers have studied spatial thinking in various STEM disciplines including geoscience, surgery, chemistry, and mathematics, and also in the K-12 setting and at the college level. Continued research into the types of spatial thinking that are required in disciplinary learning and characterize expert thinking in each domain would contribute to better theoretical understanding and educational practice. Specific questions include: How is STEM learning related to (explained or predicted by) facility with spatial thinking? Is spatial thinking different from spatial ability assessed by spatial tests? In a specific STEM discipline, what is the relationship among spatial thinking, spatial ability, and domain-specific knowledge? What is the contribution of spatial thinking, spatial ability, and domain-specific knowledge, respectively, to the mastery of each disciplinary learning? And, importantly, how can one develop curricula that effectively take scientific knowledge of spatial thinking into account to encourage students to pursue STEM careers?

### Spatial thinking as it relates to our everyday activities

Space is s fundamental component to our cognition and behavior, as it surrounds us and affords us opportunities to function adaptively. Thinking in, about, and with space characterizes (or conditions) our everyday activities. Finding one's way in the environment (cognitive mapping), communicating information in graphs and diagrams (visualization), and using space to think about nonspatial phenomena (spatial metaphors or spatialization) are major examples of our everyday spatial thinking, to name but a few. How are these everyday spatial thinking skills acquired, and if possible, instructed? Can navigation and wayfinding skills be trained, or can people's "sense of direction" be improved by training? Does the participation in spatial activities affect spatial thinking? Does self-assessment of one's spatial thinking skills affect (promote or hinder) participation in spatial activities?

Investigation of these questions, in collaboration between researchers and practitioners, will deepen our understanding of what spatial thinking is and how it relates to our cognition and behavior. We hope that the special issue fosters more research along these lines and enhances scientific and pedagogical interest in this vital domain of human cognition.
